# Joint involvement, disease activity and quality of life in pediatric Crohn’s disease – a cross-sectional study

**DOI:** 10.1186/s12969-022-00664-z

**Published:** 2022-01-29

**Authors:** Beata Derfalvi, Kriszta Katinka Boros, Doloresz Szabo, Gabor Bozsaki, Aron Cseh, Gabor Rudas, Katalin Eszter Muller, Gabor Veres

**Affiliations:** 1 Department of Pediatrics, Dalhousie University/IWK Health Centre, Halifax, Nova Scotia Canada; 2 1st Department of Pediatrics, Semmelweis University, Budapest, Hungary; 3 MR Science Centre, Semmelweis University, Budapest, Hungary; 4 Institute for Translational Medicine, University of Pécs, Pécs, Hungary; 5 Heim Pal National Pediatric Institute, Budapest, Hungary; 6 Paediatric Institute, University of Debrecen, Debrecen, Hungary

**Keywords:** Pediatric Crohn’s disease, Joint involvement, Quality of life, Arthritis, Arthralgia, Pediatric Crohn’s disease activity index

## Abstract

**Background:**

Few published data describe how joint involvement, the most prevalent extraintestinal manifestation, affects quality of life (QoL) of children with Crohn’s disease (CD). Arthritis and arthralgia rates in pediatric CD patients are reportedly 3–24% and 17–22%, respectively, but studies on pre-emptive and systematic screening of joint involvement with detailed musculoskeletal rheumatological exam are lacking. More detailed data collection on joint involvement improves our understanding of how arthropathy relates to disease activity and QoL measured by the Pediatric CD Activity Index (PCDAI) and IMPACT-III questionnaire. Our study aims were to assess joint involvement in pediatric CD and correlate it with the PCDAI and IMPACT-III.

**Methods:**

In this cross-sectional, observational study, a pediatric gastroenterologist assessed consecutively-seen pediatric CD patients at a tertiary care center. Patients were screened for prevalence of current and previous arthropathy, including arthritis, enthesitis and arthralgia. A single experienced pediatric rheumatologist evaluated detailed musculoskeletal history, joint status, and modified Juvenile Arthritis Multidimensional Assessment Reports (JAMAR). PCDAI, IMPACT-III, sacroiliac MRI, and HLA-B27 genetic testing were also completed.

**Results:**

A total of 82 (male:female, 1.2:1; age, 13.7 ± 3.2 years) patients were involved in this study. Mean disease duration at time of study was 21.6 ± 21 months; eight of the patients were newly-diagnosed. Of the 82 patients, 29 (35%) had evidence of arthritis; for 24 of those, this was revealed by physical exam during cross-sectional screening, and by prior documentation for the remaining five patients. Joint examination confirmed active arthritis in 8/24 (33%), active enthesitis in 1/24 (4%), and evidence of previous arthritis in 15/24 (62.5%) patients. Hip (41%) and knee (38%) joints were most commonly affected. Cumulative incidence of arthralgia was 48% (39/82), and 46% (18/39) of those patients had only arthralgia without arthritis, usually affecting the knee. Axial involvement was present in 10/82 (12%) patients. Joint involvement correlated with more severe CD disease activity, specifically higher PCDAI and lower IMPACT-III scores, and increased requirement for infliximab treatment. Sacroiliitis and HLA-B27 positivity were insignificant factors in this cohort.

**Conclusions:**

When a rheumatologist performed the assessment, joint involvement in pediatric CD was more prevalent than previously reported, in this cross-sectional study. Arthritis was associated with more severe CD disease activity and lower QoL.

## Background

Inflammatory bowel disease (IBD), a chronic relapsing inflammatory condition of the gut, has two major subtypes: Crohn’s disease (CD) and ulcerative colitis (UC). CD presents not only with the typical transmural, ulcerating, granulomatous lesions of the entire bowel, but with inflammation in various other organs. Different extraintestinal manifestations (EIMs) might be present in the mouth, skin, eyes, musculoskeletal or hepatobiliary systems, pancreas and, more rarely, the neurological, pulmonary or cardiovascular systems [1].

Onset of CD occurs in childhood in up to 20% of cases, and researchers describe this early start as a risk factor for severe disease [2–4]. Based on the database of the Hungarian IBD Registry Group (a nationwide incident cohort study), the incidence of pediatric CD is 4.72 in 100,000 [5]. CD occurs mostly between the ages of 10 and 17 years, with a male dominance. The rates of arthritis (the most common EIM) and arthralgia (which while not defined as a separate EIM entity is a very common complaint in children) are reported to be associated with pediatric CD at 3–24% and 14–22%, respectively [1, 6–10].

After aphthous stomatitis, the most common EIMs in patients with IBD are the arthropathies, usually occurring before or within the first year the gastrointestinal symptoms develop [7, 11]. In a recent study, arthritis was the first presenting symptom in 4% of children and adolescents with IBD. Clinical manifestations of CD-associated arthropathies vary, and include asymmetrical, transitory and migratory arthritis (pauciarticular or polyarticular), peripheral joint pain and spondyloarthropathy [12].

CD-associated arthropathies, such as sacroiliitis and arthritis and/or enthesitis, are also categorized in the heterogeneous enthesitis-related arthritis (ERA) subgroup of juvenile idiopathic arthritis (JIA), or under Juvenile Ankylosing Spondylitis if CD presents with arthritis or enthesitis of a few large joints in the extremities or with axial sacroiliitis [13]. The rates of HLA-B27 positivity and family history of a HLA-B27 associated pediatric disease are 10–60 and 20%, respectively [14–16]. In CD-related arthropathies, genetics (HLA B27 positivity, NOD2 variants), subclinical gut barrier dysfunction, dysbiosis-driven extensive innate immune ‘danger signals’ (through TLRs in macrophages), and pathological up-regulation of IL-23 and/or IL-17 axis all contribute to chronic joint inflammation [17–19].

Arthropathy significantly decreases quality of life (QoL) of adult patients suffering from IBD [20]. Similar data are missing regarding the pediatric IBD population.

We aimed with our cross-sectional study on a cohort of pediatric CD patients to determine the period prevalence (overall, including present and past) and characteristics of arthropathy by pre-emptive screening through detailed joint history and examination, sacroiliac (SI) magnetic resonance imaging (MRI) and HLA-B27 typing, to characterize the pattern and type of joint involvement in relation to disease activity, and to assess its impact on QoL using PCDAI and a QoL questionnaire (IMPACT-III).

## Methods

### Study population

We conducted an observational prevalence study comprising pediatric patients with CD seen consecutively over a 12-month period by a pediatric gastroenterologist at a single tertiary care center, the Department of Pediatrics, Semmelweis University, Budapest, Hungary. Ninety-six percent of the approached patients (82/85) agreed to be enrolled in this study, and all subjects underwent a detailed musculoskeletal history and examination by one experienced pediatric rheumatologist, who assessed objective joint involvement as evidence of arthritis, enthesitis and subjectively-reported arthralgia. During the study period, all investigators involved in the study were working fulltime in Budapest, Hungary. Applying the Porto criteria, we diagnosed CD from clinical symptoms, laboratory data, endoscopy and histopathological findings [21]. Treatment modalities at the time of the study were recorded. Additionally, a chart review on changes in management in terms of systemic medication such as DMARDs or biologic agents within 3 months of joint assessment was completed.

Patient demographics and disease characteristics, including previously documented arthritis and enthesitis, were collected from patients’ medical records. The gastroenterologist completed and reviewed the PCDAI and the IMPACT-III index, and the rheumatologist reviewed the JAMAR (Juvenile Arthritis Multidimensional Assessment Report) questionnaire at the time of the relevant visits. There was a maximum 4-week interval between the two specialists’ visits, including laboratory investigations and imaging, and no patient’s condition deteriorated so greatly in the interim as to make reassessment necessary.

We obtained study approval from the Hungarian Medical Research Council Scientific and Research Ethics Committee and written consent from all parent(s)/caregiver(s) and patients older than 7 years of age.

### Physical examination of the joints

Musculoskeletal examination was performed blinded first, to reduce the risk of examiner bias from previous and/or ongoing musculoskeletal history and symptoms, time of onset and severity of CD, or results from the study questionnaires completed by patients and their parents.

All subjects underwent a clinical examination made by a single pediatric rheumatologist focusing on 73 joints and limited to seven bilateral entheses (according to the Spondyloarthritis Research Consortium of Canada Enthesitis Index [22]).

Active arthritis was defined as joint swelling with pain and/or limited range of motion; previous “burned-out” arthritis was defined as severely restricted range of motion with/without deformity [23]. Enthesitis was defined as tenderness by palpation (examiner applying enough pressure with the thumb to blanch the nail bed) with/without swelling [24]. Arthralgia was defined as localized, not exercise-related, persistent joint pain at rest, with no evidence of arthritis. All patients or caregivers were able to report pain reliably and accurately. Patients with arthritis in at least one joint as well as arthralgia in another joint(s) was classified as having arthritis.

### Laboratory investigations and imaging

Basic laboratory parameters including CRP and complete blood count were determined at the time or within 4 weeks of joint assessment. SI MRI (axial 3D WAVE, T2 SPAIR, 3D DWIBS, sagittal and coronal T2 sequences by Philips Achieva 3 Tesla) as added sequences targeted to SI joint were evaluated during MR enterography (MRE) (*n* = 62). Molecular genetic testing for HLA-B27 (*n* = 58) was also performed. A radiologist with experience in pediatric and skeletal imaging, blinded for physical joint exam results, interpreted the MRI findings for evidence of SI joint inflammation. Inflammatory sacroiliitis was diagnosed according to ASAS 2009 criteria, in particular the presence of bone marrow oedema in the subchondral or peri-articular compartment of the SI joint [25]. Additionally, structural changes such as erosions, fatty degeneration, sclerosis, and ankylosis as indicative of previous active inflammation were included in the analysis.

### Questionnaires

#### Jamar

Questionnaires completed by the parent and by the child him/herself (if older than 7–8 years) were used to assess functional joint status, joint pain, joint disease, morning stiffness and joint disease activity. Only responses to questions 1–4, 6 and 7 of the translated, cross-culturally adapted and validated, minimally modified version of JAMAR [26, 27], were analyzed in this study. Modification included specifying joint pain (questionnaire questions 2 and 6) and joint involvement (questions 6 and 7).

#### IMPACT-III (HR)

The patient him/herself (if older than 7–8) completed the IMPACT-III questionnaire, a disease-specific, health-related QoL instrument containing 35 questions encompassing six domains: bowel symptoms (six concerns), systemic symptoms (three concerns), emotional functioning (seven concerns), functional/ social impairment (16 concerns), body image (three concerns), and tests/treatments (three concerns). For each question, answers were assessed using a Likert-type scale with five responses, resulting in full scores ranging from 35 to 175. Higher scores indicate a good QoL, lower scores indicate the disease has negatively affected patient QoL [28].

#### PCDAI

PCDAI value was used for monitoring disease activity. PCDAI includes history items, findings about the physical examination (including extraintestinal manifestations) and laboratory tests (Supplement). PCDAI scores range from 0 to 100; the higher score reflects more active disease. PCDAI score < 10 indicates inactive disease, between 10 and 30 a mild disease, and if PCDAI score is ≥30 we speak about an active disease [29, 30].

### Statistical analyses

All statistical analyses were performed by a biomedical statistician. Calculations and data analysis were performed using IBM SPSS Statistics (version 21.0, IBM, Armonk, NY, USA). Descriptive measures (medians, means, ranges, and SE) were calculated for continuous variables (i.e., age, gender and clinical disease activity indices such as laboratory parameters, use of medication, scores of disease activity indices), while frequencies were calculated for categorical variables (i.e., different types of joint involvement), along with 95% confidence intervals (CIs) for means and proportions. Variables were examined for normal distribution with the Kolmogorov-Smirnov test and Dunn’s multiple comparison test was used in case data were not normally distributed. Means were compared using Student’s *t*-test. Categorical variable comparisons were performed using Fisher’s exact test, chi logistic regression, and chi square test to examine any possible association between clinical disease activity indices (PCDAI, CRP, PLT, IMPACT-III; all continuous variables), and joint involvement such as arthritis, joint contractions and arthralgia on examination, in documented history or on JAMAR and biological therapy (as a categorical variable). A *p* value of < 0.05 was considered significant.

## Results

A total of 82 patients were involved in this study. Eight of 82 patients were “newly-diagnosed” with IBD within 4 weeks prior to rheumatology assessment. MRE was performed in 62; molecular genetic testing for HLA-B27 was performed for 58 patients. Table 1 shows patient demographic and clinical data. At disease onset, two patients were less than 2 years old (had infantile IBD), three were 2–6 years old (had very early onset IBD (VEO-IBD)) and 14 were between 6 and 10 years old (had early onset IBD). The remainder were 10–18 years of age. Increased CRP and thrombocytosis reflect chronic active inflammation in this cohort. Eighty-nine percent (73/82) of patients were on systemic immunosuppressive medication for their CD, including only three who received systemic medication also for arthritis.

**Table 1:** Demographic and clinical data of patients studied

Parameter	Patient value/description
Gender ratio (male:female)	1.2:1
Age at time of study (in years mean ± SD)	13.7 ± 3.2
Age at onset of Crohn’s disease (in years mean ± SD)	12.2 ± 3.6
Disease duration (in months mean ± SD)	21.6 ± 21
Disease duration median number of months	15
Overall scores on PCDAI (mean ± SD)	11.5 ± 14.2
Overall scores on IMPACT-III index (mean ± SD)	103.5 ± 30
CRP (mg/L, mean ± SD)	11.8 ± 19.3
PLT count (10^9^, mean ± SD)	422 ± 145
Medications at time of study	
sulfasalazine/mesalamine	70/82 (85%)
azathioprine	58/82 (70%)
infliximab	16/82 (20%)
methylprednisolone	16/82 (20%)
budenoside	7/82 (9%)
methotrexate	5/82 (6%)
NSAID	3/82 (4%)

**Fig. 1:**
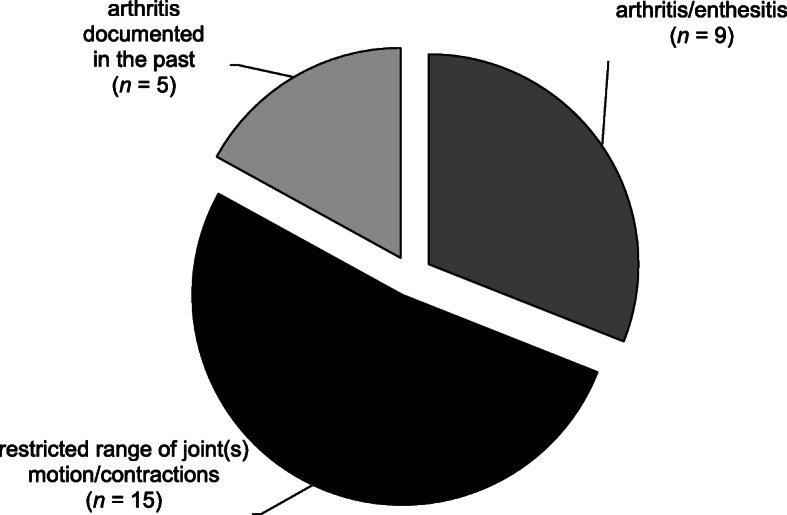
Prevalence of joint involvement by objective measurement in our pediatric CD cohort. Legend: Prevalence of joint involvement (arthritis, enthesitis, restricted range of motion) by objective measurement (physical examination or documented history). Conditions occurred as indicated in a total of 29 of 82 patients studied (35%)

Thirty-five percent (29/82) of the pediatric CD patients had objective arthritis. Arthritis was diagnosed in 24/29 based on physical examination (point prevalence), and 5/29 patients had a remote history of documented active arthritis (period prevalence) (Fig. 1).

Of the 24 patients with arthritis, only one had active enthesitis at the time of examination. Eight of these 24 patients (33%) had active arthritis indicated by swollen joint(s) with or without stress pain, including four patients who also had a restricted range of motion or contraction in one or more other joints, suggestive of longstanding or previous arthritis. The remaining 15 patients showed evidence of previous arthritis on joint examination indicated by a severely restricted range of motion with or without deformity in one or more joints. Hip (12/29 children) and knee (11/29 children) joints were most commonly affected. In terms of arthritis, 41% (12/29) had axial, and more than half had peripheral presentation; 17% had monoarticular, 21% had oligoarticular and 21% had polyarticular involvement. Sixty-two percent (18/29) had arthritis at the onset of CD.

Seventy-one percent (17/24) of patients with arthritis on exam had joint complaints at the time of evaluation; all had arthralgia and three of them complained about morning stiffness as well.

The frequencies, as determined by physical examination or patient history, at which individual joints were affected and the characteristics of affected joint inflammation are depicted in Table 2.

**Table 2:** Distribution of joints with active or previous arthritis found by physical examination or documented in medical chart

	Condition revealed by physical examination during current study		Documented in patient’s chart
Joint	Active arthritis (and/or enthesitis) (***n*** = 9 patients)	Restricted range of motion (***n*** = 15 patients)	Arthritis (and/or enthesitis) (***n*** = 5 patients)
Shoulder	1/9		
Elbow	1/9		
Hand	1/9	3/15	
Lumbosacral	4/9		1/5
Hip	1/9	11/15	
Knee	4/9 (1/9)	6/15	1/5 (1/5)
Ankle	1/9	2/15	3/5
Foot	1/9	1/15	2/5

The cumulative incidence of arthralgia during the entire course of the disease was as high as 48% (39/82 patients); however, fewer than half (18/39; 22% of the total number of patients) had only arthralgia, without arthritis on exam. Distribution of arthralgia in 18/82 patient history or JAMAR, is shown in Table 3.

**Table 3:** The most frequently subjectively-affected joints

Joint	Frequency of arthralgia based on history (*n* = 18 patients)
Shoulder	1/18
Elbow	0/18
Hand	0/18
Lumbosacral	2/18
Hip	2/18
Knee	14/18
Ankle	6/18
Foot	0/18

The most frequently affected joints by subjective joint involvement (i.e., arthralgia with or without morning stiffness) in 18/82 patients were the knee (14/18), followed by ankles (6/18) by history or JAMAR.

Axial involvement was present in 10/82 (12%) of the patients, less frequently than in adults. Sacroiliitis was documented in one patient’s medical record by MRI imaging, and was suspected in another three by positive Mennell’s sign (sacroiliac-pain provocation test in early active sacroiliitis) on physical examination and lumbosacral pain in history, but was unconfirmed by SI MRI. SI MRI as added sequences to MRE was done in a subset of patients when MRE was a part of the standard of care, and no patient among those without low back or hip pain (55/62, 89%) had subclinical sacroiliitis.

Spondylarthritis involving the lumbosacral spine by limited range of motion in the Schober’s test was positive in one patient, and another two complained about lower-back pain and morning stiffness. Overall HLA-B27 positivity was 10% of patients, and frequencies were similar within the axial (10%) or peripheral (11%) arthritis groups and in patients without arthritis (9%). The only tender enthesis in the study was identified in a single patient, at the patellar ligament insertion at the inferior pole of the patella.

Two patients with arthritis had also psoriasis on physical exam and another one had a first degree relative with psoriasis, similarly to seven additional patients without arthritis.

**Fig. 2:**
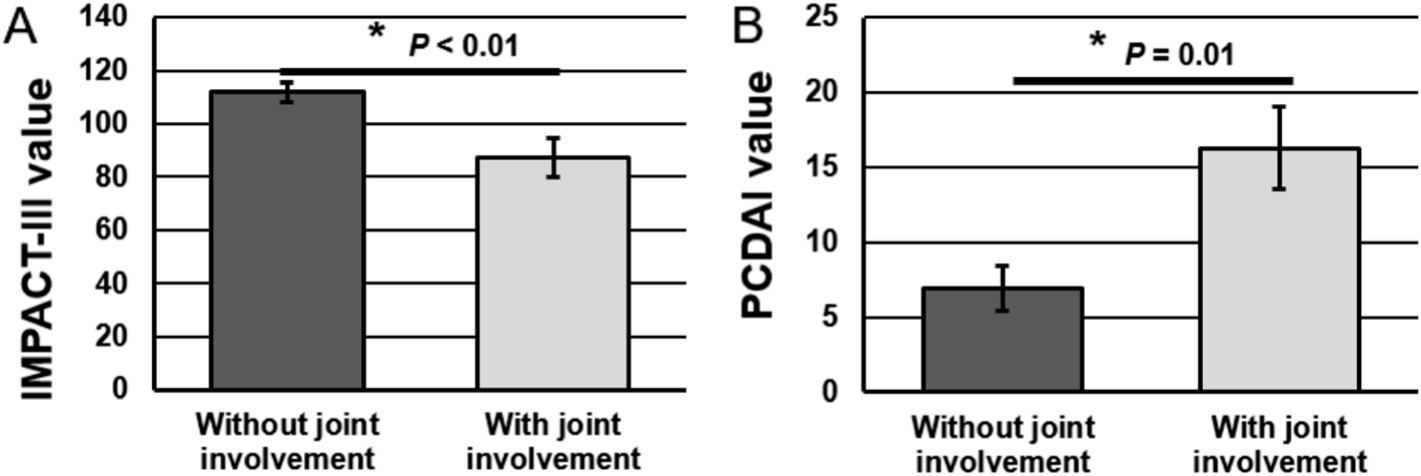
IMPACT-III **a** and PCDAI **b** indices in pediatric CD patients without or with joint involvement (arthritis, enthesitis and arthralgia). Values are means ± SE (*n* = 82 patients)

Figure 2A shows a significant association (*p* < 0.01) between joint involvement (arthritis and/or arthralgia) and lower QoL, as measured by the IMPACT-III. There is a significant association between joint involvement and PCDAI. Furthermore, IMPACT-III values negatively correlated with PCDAI scores in subgroup of pediatric CD patients who had arthritis (*r* = − 0.64, *p* = 0.0024), but not without arthritis (*r* = − 0.35), suggesting that the lower QoL was associated with joint involvement, and did not originate from GI disease. Cumulative frequencies of arthritis and/or arthralgia (but not axial or peripheral joint disease when examined separately) correlated with more aggressive disease, reflected by higher PCDAI scores at the time of the study (*p* = 0.01) (Fig. 2B).

Overall, arthritis was significantly more common (according to logistic regression analysis) in patients requiring biological therapy, and patients with arthritis detected at the onset of their CD (*n* = 18) received more aggressive treatment, including the TNF-inhibitor biologic infliximab. Active arthritis or other joint involvement at the time of joint assessment, however, was not more common in patients treated with infliximab. Details of drug therapies are presented in Table 1.

There were no correlations with objective joint involvement, including active or documented arthritis, or subjective signs of joint involvement, such as arthralgia, morning stiffness and restricted range of motion in history, with age, gender, PCDAI at onset of CD, the follow-up time from diagnosis, CRP or platelet count at examination. Objective joint involvement was uncorrelated with IBD, spondylarthropathy or psoriasis in first- or second-degree relatives. There was no significantly higher incidence of active arthritis or other joint involvement in patients examined in the first 6 or first 12 months after onset of CD compared to those with longer follow-ups.

Rheumatology evaluation led to changes in management in terms of systemic medication in 25% of patients (6/24); three were started on biologic agents (infliximab) within 3 months of rheumatological exam, due to joint involvement while having active IBD disease on additional therapies, two started NSAID, and one started corticosteroid within 2 months of the joint assessment.

## Discussion

Approximately 25% of all IBD cases begin in childhood, causing a severe form of this life-long disease with childhood-specific morbidity such as poor bone density, growth failure and delayed puberty [5]. Increased disease severity at baseline was associated with the occurrence of any EIM including arthralgia in a large cohort of pediatric CD patients [6]. Presence of extraintestinal manifestations overall had a negative impact on scores from the emotional and social functioning domains of the IMPACT-III questionnaire in a single study [31]. Studies on recognized associations between joint involvement and disease severity later during the course of disease are inconclusive in adult and pediatric CD patients; similarly, studies analyzing the type of joint involvement in pediatric CD and QoL are markedly rare compared to adult studies [6, 11, 32–34].

Reliable reports of pediatric CD-associated arthropathies are difficult to generate. Most publications report data from charts analyzed retrospectively, where clinically-obvious arthritis was present, mainly detected by the gastroenterologist in charge. Since CD-associated arthropathies also belong to the very heterogenous ERA subgroup of JIA, children might be diagnosed with JIA while IBD remains undiagnosed. In one-fifth of adult patients, peripheral arthritis appeared before IBD (including colitis) was diagnosed [35]. Of patients in our cohort who had arthritis any time during their CD, 62% (18/29) had arthritis at the onset of CD, and this predicts a more severe disease course. To obtain earlier diagnoses, every child presenting with acute or chronic arthritis should be assessed for IBD at least by specific personal history of gastrointestinal symptoms as well as a family history of IBD [12, 36].

Because of the transient and fluctuating presence of oligoarthritis and its dramatic response to corticosteroid and other treatment for exacerbations of the IBD, we can only estimate the incidence or prevalence of CD-related pediatric arthropathies. Also, detailed joint examination is unusual during a routine gastrointestinal examination. As well, arthritis and arthralgia tend to be combined in the CD literature, making it impossible to distinguish the presence of true arthritis.

Inflammatory joint involvement is more common (24%) in newly-diagnosed IBD [37]; our study, however, found no significantly higher incidence of joint involvement in children at the onset or early in the course of their CD compared to those with longer follow-up. There were only four newly-diagnosed patients included in the study, which could potentially explain the negative finding when comparing frequency of arthritis between newly-diagnosed and established CD compared to previous studies.

Sacroiliitis is estimated as at least 30 times more common in IBD patients than among adults in general, with a pooled prevalence of 21% [38]. Axial involvement was present in 10/82 (12%) of our pediatric patients, less frequently than in adults. Sacroiliitis found by physical examination or identified as lumbosacral pain with or without morning stiffness in history was not confirmed by SI MRI. We detected no subclinical sacroiliitis based on MRI combined with MRE, although the highly sensitive STIR sequences were not analyzed. Instead, a conventional MRI exploration, dedicated to SI joints, and taking an extra 10 min during imaging, was added to the MRE. This approach allows accurate differentiation between intestinal manifestations and axial symptoms, contributing to an earlier diagnosis of axial spondylarthritis in IBD patients [39, 40]. SI pain, misdiagnosed due to radiating pain in the colon, might also be incorrectly interpreted as IBD-related. In our cohort HLA-B27 positivity was not more prevalent in the subgroup of patients with joint involvement. In fact, similarly to the healthy population, only 10% of our patients even carried the HLA-B27 allele, which might explain the low incidence of enthesitis in the cohort. This concurs with other studies, as HLA-B27 in the pediatric IBD population is generally not higher than in the general population [32], despite the fact that 12% of our patients had psoriasis or a first-degree relative with psoriasis, which is another HLA-B27 associated disease.

Enthesitis was uncommon in our cohort, although it is reported elsewhere as the most common EIM in pediatric CD with a 21% prevalence; it is also common in adults (7–50% prevalence) [41–44]. This could be explained by the younger age group in our study compared to the relatively small and older group reported [42].

The therapeutic modalities in CD patients with related arthropathies, including local steroid injections, physiotherapy and anti-TNF therapy (infliximab), overlap with those in JIA. In CD, however, therapies are modulated according to the associated features (uveitis, psoriasis, pyoderma gangrenosum, colitis) and with preferential use of selective COX-2 inhibitors to reduce exacerbation of IBD due to NSAID and sulfasalazine as initial DMARD, as were used in our patients [32]. Joint assessment in our study increased our attention to symptom control, which led to changes in management in terms of systemic medication in 25% of patients.

Our prevalence study is limited in that our findings reflect joint involvement in a cohort heterogeneous in terms of disease duration and severity as well as treatment modalities. Another limitation is that CD is a heterogeneous group of different diseases, and several as distinct entities might be associated with arthritis, especially monogenic immune dysregulatory diseases, also known as inborn errors of immunity [45]. According to the Paris Modification of the Montreal Classification system [46], in VEO/EO-IBD cases, contribution of genetic factors in the pathogenesis is high, and the disease is characterized by a more severe phenotype: a rapid progression with complications and resistance to conventional therapies. Based on patient age at CD onset in our cohort, a significant proportion (19/82; 23%) would be classified in special subgroups of IBD. Up to 50 monogenic inborn errors of immunity may underlie VEO/EO-IBD, but identifying molecular defects in our patients and their association with arthritis is beyond the scope of this study [47]. Since all but three of the consecutively-seen and approached patients agreed to be enrolled into this study, recruitment bias (whereby parents of children with joint complaints could be more likely to consent to the study compared to parents of children without joint complaints) is unlikely, and thus does not pose a potential limitation.

## Conclusion

In conclusion, our study is the first in which a pediatric rheumatologist systematically and pre-emptively screened and analyzed joint involvement in pediatric patients with CD, and which included SI MRI and HLA-B27 typing. In addition to the point prevalence data, use of chart review for past documented arthritis and the arthritis-specific questionnaire (modified JAMAR) allowed us to estimate period prevalence data, which can be incorporated into the calculations in cross-sectional studies. In addition, parallel estimations of QoL and PCDAI were determined. Our results showed that when assessment is done by a pediatric rheumatologist, the prevalence of joint involvement in pediatric CD is higher than previously reported. Joint involvement including arthritis and arthralgia is associated with more severe disease, as reflected by higher PCDAI scores and lower QoL.

Although the majority of pediatric patients with CD who were identified as having arthritis on exam also had arthralgia at the time of evaluation, almost one-third of them had arthritis without joint complaints; therefore, a thorough joint exam by the assessing gastroenterologist is recommended.

Arthropathic conditions in pediatric CD are very common and often underestimated, despite association with a significantly higher impairment of QoL. Pre-emptive and systematic screening of joint involvement is essential, and helps identify a parameter that predicts a severe disease course early, thus optimizing therapeutic decision-making and personalized treatment.

## Data Availability

The datasets used and analysed during the current study are not publicly available, as they contain identifiable information, but are available from the corresponding author on reasonable request.

## Abbreviations

CD: Crohn’s disease
CRP: C-reactive protein
EIMs: Extraintestinal manifestations
ERA: Enthesitis-related arthritis
IBD: Inflammatory bowel disease
JAMAR: Juvenile Arthritis Multidimensional Assessment Report
JIA: Juvenile idiopathic arthritis
MRE: Magnetic resonance enterography
MRI: Magnetic resonance imaging
NSAID: Non-steroidal anti-inflammatory drugs
PCDAI: Pediatric Crohn’s Disease Activity Index
PLT: Platelet
QoL: Quality of life
SI: Sacroiliac
UC: Ulcerative colitis
VEO-IBD: Very early onset inflammatory bowel disease
